# Reliability of judging in Olympic breaking at the 2024 Paris games

**DOI:** 10.3389/fpsyg.2025.1593158

**Published:** 2025-12-03

**Authors:** Nahoko Sato

**Affiliations:** Department of Physical Therapy, Faculty of Rehabilitation Sciences, Nagoya Gakuin University, Nagoya, Japan

**Keywords:** breakdance, dance, Olympics, hip-hop, aesthetic sports, judge, validity, bias

## Abstract

**Introduction:**

Although breakdancing was adopted as an Olympic sport at the 2024 Paris Olympics, difficulty of techniques and objective evaluation criteria for each technique were not clearly defined. The reliability of the evaluation system for breaking at the 2024 Olympics has not yet been analysed in the literature. This study reviews research on judging in other aesthetic sports and aims to evaluate the reliability of the scoring results for breakdancing at the 2024 Paris Olympics.

**Methodology:**

The results of the 2024 Paris Olympics were extracted from the official Olympics website. The competition was conducted in a one-on-one battle format. Using a slide bar, the judges evaluated which dancer performed better in five categories: technique, vocabulary, originality, execution, and musicality. Absolute deviations from the final score for individual judges were calculated as measures of bias for validity analysis. The reliability of the evaluation was assessed using the intra-class correlation coefficients and Kendall’s W.

**Results:**

This study found that the reliability of the judges’ scores was equivalent to that of DanceSport and hip-hop dance competitions and was considerably lower than that of artistic gymnastics competitions. Moreover, the originality category demonstrated good reliability, while the other four categories showed poor reliability. The judges were aware of the characteristics of breakdancing, where the winner was determined by the impressions and excitement of the audience, and this may have led to more reliable scoring in the artistic rather than in the technical category.

**Discussion:**

These results will contribute to developing a more reliable evaluation system for hip-hop dance competitions.

## 1 Introduction

Hip-hop dance originated in the late 1960s in New York, United States, primarily among young Hispanic and African-American men (Craine and Mackrell, 2010). In recent decades, hip-hop dance competitions have been held worldwide (Hip Hop International, 2023a). Breaking—often referred to as ‘breakdance’ in the media—is a style of hip-hop dance that was introduced at the 2018 Youth Olympic Games and subsequently adopted as an Olympic event at the 2024 Paris Olympics. As hip-hop dance has evolved into a globally recognised aesthetic sport, the performance evaluation system should be highly reliable, with its criteria understandable by the audience. Many studies on the evaluation systems for other aesthetic sports, such as gymnastics and figure skating, which have been adopted as Olympic events for over 100 years, can be referenced in developing a reliable evaluation system for hip-hop dance. This study reviews the existing research on judging in other aesthetic sports and assesses the reliability of the scoring results for breakdancing at the 2024 Paris Olympics.

### 1.1 Literature review

Judging in aesthetic sports relies heavily on the subjective evaluations of experienced judges. Issues and biases within the evaluation systems for aesthetic sports have been investigated by analysing past competition data (Bučar et al., 2012; Leskošek et al., 2010; Lockwood et al., 2005; Pajek et al., 2014). Reputation bias has been reported in judging figure-skating and diving, wherein judges accorded favourable ratings to recognised athletes (Findlay and Ste-Marie, 2004; McGee, 2019). Memory-influenced bias was found in gymnastics judging (Ste-Marie et al., 2001), as the memory of prior performances of athletes was reported to affect judges’ evaluations. Judges’ experience is an important factor influencing evaluation reliability (Flessas et al., 2015; Heinen et al., 2012; Mack, 2019; Yee and Hoong, 2013). In gymnastics, Flessas et al. (2015) found that international judges could detect errors better than national and novice judges. Judges’ view angles of performance can influence evaluations in gymnastics. A previous study showed that the most reliable view angle was from the front of the performance and that deviations of the judges’ positions from that position resulted in lower reliability of their scores (Dallas et al., 2011). The evaluation systems for these sports were revised several times to achieve more reliable evaluations, and support tools, such as real-time evaluation systems and video clips, were introduced (International Skating Union, 2024; Pajek et al., 2011).

Among aesthetic sports, the reliability of the scoring in gymnastics is reported to be very high, and it may become an ideal model for the evaluation system of hip-hop dance. Gymnastics was adopted in the first Olympics in 1896. The ‘Code of Points’ evaluation system used in artistic gymnastics was first created in 1949 and was designed to support objective performance evaluations (Atiković et al., 2011). In artistic gymnastics, the difficulty and execution scores evaluate exercise content and execution, respectively. The Code of Points defines the difficulty values and kinematic criteria for all performance-related skills. Atiković et al. (2011) reported that the reliability of judges’ evaluation scores was very high (Cronbach’s alpha ranged from 0.94 to 0.98) in men’s artistic gymnastics at the World Championship 2009. High reliability was also reported in evaluation scores in the 2011 European championship in Berlin (Pajek et al., 2013) and the 2009 World University Games (Bučar et al., 2012; Leskošek et al., 2010). On the other hand, Pajek et al. (2014) analysed artistry judging at the World Championship 2011 competitions and reported low inter-rater reliability (average correlation coefficient among all pairs of judges was 0.6), with large disagreement in artistry deductions. There is ample opportunity to replicate studies in artistic gymnastics to investigate crossover opportunities in hip-hop dance to support the evolution of hip-hop evaluation reliability.

Figure skating, like gymnastics, has been an Olympic sport for over 100 years. Lockwood et al. (2005) reported high reliability of the judges in figure skating scores at the 2002 Winter Olympics, demonstrating higher reliability in the technical than in the artistic score. However, in response to the scandal that occurred at the 2002 Olympics, the International Skating Union (ISU) initiated the implementation of the International Judging System (IJS; Looney, 2012). The IJS attempts to reduce judges’ discretion by making scoring more objective. In the IJS, the final score combines the scores for technical elements and programme components, and accounts for deductions based on obvious criteria, such as falls. After the 2002 Olympics, the ISU also decided that judges’ scores would be reported anonymously and selected randomly for the calculation. The information to investigate the reliability and validity of judging on IJS was inadequate because of this anonymisation and randomisation.

Dance competitions, such as dance sports and hip-hop, unlike gymnastics, are conducted without defined difficulty levels for the techniques and clear evaluation criteria for each technique. Dance sport has a longer history than hip-hop dance, with its first world championship being held in 1922. During its history, the evaluation system has also been revised to achieve greater reliability, therefore it may apply to hip-hop dance as well. The World DanceSport Federation introduced a new judging system in 2013 (World DanceSport Federation, 2022). Until then, in the preliminary rounds, judges would mark and select several dance couples to advance to the next round. In the final round, judges were required to rank each couple. Thus, the previous judging system used relative evaluation. The new judging system introduced absolute evaluation, including technical and artistic sections with two categories each. Premelč et al. (2019) investigated the reliability of the new judging system in DanceSport and reported that the overall judging correlation was 0.48, with the artistic section showing a slightly lower coefficient than the technical one. This value was quite low compared to that of gymnastics competitions (Atiković et al., 2011; Bučar et al., 2012; Leskošek et al., 2010; Pajek et al., 2013). This may be because the difficulty and kinematic criteria for evaluating all the techniques in gymnastics are defined in the Code of Points, but difficulty levels and evaluation criteria are not specified for DanceSport. Judges must evaluate at least six couples dancing simultaneously, which also contributes to the low reliability of their scoring.

Hip-hop dance competitions face similar challenges in evaluation reliability due to the lack of standardised difficulty levels and objective criteria. There are two types of hip-hop dance competitions: championships and battles (Hip Hop International, 2023b). In the championships, dance teams composed of multiple dancers perform a choreographed routine and are evaluated. The dance team with the highest score wins. In the battle, the dancers basically perform improvised performances one-on-one in turn, and the judges evaluate which dancer is better. In hip-hop dance championships, judges must evaluate performance in several categories, such as musicality and synchronisation (Hip Hop International, 2023b). The final ranking is determined by the total score in each category. Sato (2022) analysed the reliability of the evaluation results of hip-hop dance championships and reported that Kendall’s W values ranged from 0.319 to 0.681. This indicates very low values compared with artistic gymnastics competitions (Atiković et al., 2011; Bučar et al., 2012; Leskošek et al., 2010; Pajek et al., 2013). In rhythmic gymnastics, the degree of difficulty and kinematic criteria for correct movement for all the techniques are included in the Code of Points. However, the difficulty of the techniques and criteria for evaluating movements in hip-hop dance are not clearly defined, which implies that the performance evaluation criteria may differ depending on judging.

Breaking, as one hip-hop dance genre, has unique artistic and cultural characteristics that distinguish it from other aesthetic sports. It has evolved from a form of street expression to a globally recognised competitive discipline, reflecting both athletic and creative values (Pop, 2023). Within this sportification process, creativity and originality are considered central judging elements; however, a lack of shared understanding of creativity among judges can compromise evaluation reliability (Yang et al., 2022). Furthermore, recent research has emphasised the importance of establishing standardised and transparent criteria for evaluating and researching Olympic breaking, reflecting growing attention to methodological clarity in the field (Yang and Whatman, 2025). These aspects highlight that breaking judges must consider not only technical execution but also expressive and cultural dimensions.

No prior study has investigated the reliability of judging in one-on-one breaking, the format adopted in the 2024 Paris Olympics. Judges used a slide bar to evaluate which dancer was superior in five categories: technique, vocabulary, originality, execution, and musicality (World DanceSport Federation, 2024) Each of the five categories accounted for 20% of the final score, and the dancer with the highest total score was the winner. Although the major focus areas to be evaluated were clearly indicated in each category (Table 1), specific evaluation criteria were not defined. This study aimed to evaluate the reliability of the scoring results for breakdancing at the 2024 Paris Olympics.

**Table 1:** The five categories used in judging at the 2024 Paris Olympics.

Category	Major focus areas
Technique	Athleticism/form (lines, angles, shapes)/body control/dynamics/spatial awareness
Vocabulary	Variation/quantity of moves/repeat
Execution	Cleanliness/minimal to no slips, crashes, or falls/consistency of flow/composition/storytelling (narrative)
Musicality	Rhythm/texture/synchronicity/accenting
Originality	Improvisation/innovation/spontaneity/personality/response

## 2 Methods

The results of the men’s breaking competition at the 2024 Paris Olympics were extracted from the official Olympics website (Paris 2024, 2024). This study used all results from the preliminary to the final rounds. A total of 32 battles were analysed, involving 16 athletes: 24 preliminaries, 4 quarterfinals, 2 semifinals, 1 third-place battle, and 1 final. In the preliminaries, the 16 athletes were divided into four groups of four, within which they competed in round-robin battles; the top two dancers from each group advanced to the quarterfinals. Each battle consisted of two rounds in the preliminaries and three rounds from the quarterfinals onwards (the final phase).

The five categories of technique, vocabulary, originality, execution, and musicality were allocated 20% each, for a total of 100%. The major focus areas for the five categories are shown in Table 1 (World DanceSport Federation, 2024). The judges moved the slide bar to the dancer who was better in each category and assigned points. In the case of a tie in a category, 10% of each would be earned. In this study, the percentages allocated to each category were used as the points obtained, and the mean value of the scores assigned by the nine judges was used as the final for each category.

Absolute deviations from the final score for individual judges were calculated as measures of bias for validity analysis (Bučar et al., 2012). For both, the two-way random effects (consistency) and fixed effects (agreement), the reliability of the evaluation was assessed using the intra-class correlation coefficients (ICCs) for the mean values of single and nine raters (Premelč et al., 2019). Kendall’s W (Kendall’s coefficient of concordance) was also tested. The ICCs were interpreted as follows: less than 0.40 indicated low reliability, 0.4 to 0.75 indicated adequate reliability, and over 0.75 indicated excellent reliability. Kendall’s W values of less than 0.40 were considered poor, 0.40 to 0.50 were considered moderate, 0.50 to 0.70 were considered good, and values over 0.70 were considered excellent. All data were analysed using SPSS Statistics software (version 25.0; SPSS Inc., Chicago, IL, United States).

## 3 Results

Table 2 shows the descriptive statistics and correlations of the judges’ scores. Regarding score validity, absolute deviation from the final score was largest for technique and smallest for originality. Regarding the correlation between the scores of the individual judges and the final score, which was the mean of the scores of the nine judges, originality demonstrated the highest correlation values.

**Table 2:** Performance of individual judges.

	Mean			SD			Lowest marks			Highest marks			Absolute deviation		Correlation with 5-judge average (r)	
Category	Mean	Max	Min	Mean	Max	Min	Mean	Max	Min	Mean	Max	Min	Max	Min	Max	Min
Technique	10.00	10.00	10.00	2.19	3.90	1.28	5.33	7.80	2.20	14.67	17.80	12.20	**2.99**	0.80	0.75	0.47
Vocabulary	10.00	10.00	10.00	2.24	3.29	1.01	5.49	8.00	2.20	14.51	17.80	12.00	2.34	0.94	0.83	0.36
Originality	10.00	10.00	10.00	2.36	3.66	1.36	4.51	7.40	0.00	15.49	20.00	12.60	**2.20**	0.88	0.87	0.51
Execution	10.00	10.00	10.00	2.29	3.30	1.33	4.07	8.00	0.00	15.93	20.00	12.00	2.25	0.94	0.83	0.41
Musicality	10.00	10.00	10.00	2.46	4.97	1.26	5.33	7.40	1.20	17.89	47.80	12.60	2.82	0.80	0.77	0.51

Bold values indicate the largest and smallest absolute deviations from the final score, corresponding to the technique and originality categories, respectively.

The single-measure ICCs for both absolute agreement and consistency demonstrated good reliability for originality, but poor reliability for the other four categories (Table 3). Kendall’s W coefficient also showed results similar to those of ICCs, with a moderate reliability coefficient of 0.570 for originality (Table 3).

**Table 3:** Coefficient of reliability (ICC and Kendall’s W) for the five judging categories.

Category	ICC								Kendall’s W coefficient	
	Absolute agreement				Consistency					
	Single	95% CI	Average	95% CI	Single	95% CI	Average	95% CI	W	*p*
Technique	0.206	0.154–0.270	0.700	0.621–0.769	0.205	0.153–0.268	0.699	0.619–0.767	0.322	0.000
Vocabulary	0.290	0.230–0.360	0.786	0.729–0.835	0.288	0.229–0.358	0.785	0.728–0.834	0.364	0.000
Originality	**0.452**	0.386–0.524	**0.881**	0.850–0.908	**0.450**	0.384–0.522	**0.880**	0.849–0.908	**0.570**	0.000
Execution	0.291	0.232–0.361	0.787	0.731–0.836	0.290	0.230–0.359	0.786	0.729–0.835	0.403	0.000
Musicality	0.277	0.218–0.346	0.775	0.716–0.826	0.276	0.218–0.345	0.774	0.715–0.826	0.464	0.000

Bold values indicate the highest reliability coefficients (ICC and Kendall’s W) for the originality category.

For reference, reliability values calculated separately for the preliminary and final phases are presented in Supplementary Tables S1, S2, showing similar trends across both competition formats.

## 4 Discussion

This study examined the reliability of the scores assigned by judges at the 2024 Paris Olympics breaking competition. As this was the first inclusion of breaking as an Olympic event, the findings provide fundamental data for improving the reliability of judging it as a competitive sport. The main finding was that the reliability of judges’ scores was comparable to that in DanceSport and hip-hop dance championships but considerably lower than in artistic gymnastics competitions. In addition, although the competition format differed between the preliminary and final phases, the reliability estimates remained comparable, suggesting that judging consistency was not strongly influenced by the event structure. The technique category, which was included in the technical category, showed large variation and low reliability, whereas the originality category, which was included in the artistic category, demonstrated little variation and high reliability.

One reason for the low reliability of breaking judges’ scores was the lack of defined criteria for evaluating performance and the degree of difficulty of moves. In artistic gymnastics, performance is evaluated using an objective scoring system, based on the rules detailed in the Code of Points, and several studies have shown that high reliability of judges’ scoring (Atiković et al., 2011; Bučar et al., 2012; Leskošek et al., 2010; Pajek et al., 2013). Conversely, while breaking has defined categories and key focus areas for evaluation, the detailed kinematic criteria for each technique and the criteria for evaluating each level along a scale are not explained. These deficiencies are also common to hip-hop dance championships and dance sports. As the reliability of breaking scores in Paris 2024 was equivalent to that of DanceSport and hip-hop dance championships (Premelč et al., 2019; Sato, 2022), it can be inferred that the lack of defined evaluation criteria impacts reliability to a greater degree than does the difference between absolute and relative evaluation.

While technique had a large deviation and low reliability, originality had a small deviation and high reliability. A previous study that analysed the reliability of judges’ scores in hip-hop dance championships showed that technical categories were more reliable than artistic categories (Sato, 2022). Similar results were found in figure skating (Lockwood et al., 2005), gymnastics (Pajek et al., 2014), and dance sports (Premelč et al., 2019), which contradict the results of the present study. The characteristics of breaking are strongly connected with the creative expression of identity, emotion, and artistic sensitivity (Pop, 2023; Yang et al., 2022). It is believed that the winner is decided by the impression formed by the excitement of the audience. The judges who assessed performances at the Paris Olympics were aware of these characteristics, which may have resulted in more reliable and valid scores in the artistic categories than in the technical ones. In addition, the higher reliability of originality may be partly explained by methodological factors. Specifically, compared with the more technical categories—particularly technique and vocabulary—the evaluation criteria for originality might have been more clearly defined and less ambiguous, as the technical categories encompass a broader and more complex range of movements in breaking. Furthermore, unlike hip-hop dance championships, dance sports, and gymnastics, which are judged using absolute evaluation, breakdancing at the Paris Olympics was judged using relative evaluation. This suggests that relative evaluation may be more suitable for judging artistic categories. It may be difficult to use an absolute evaluation system to evaluate artistic categories, as there is no single parameter that can be used to evaluate them.

Since hip-hop dance has been introduced into international sporting events through breaking, a reliable evaluation system is required to further develop it into an aesthetic sport. Hip-hop dance competitions should be conducted in a manner that explains the judgement criteria in detail, allowing the audience and dancers to understand the results of the judgement. First, to establish a reliable evaluation system, biases that might affect judges’ assessments, such as seat position and view angle of the performance, the evaluation categories to be scored, and how the final scores are calculated should be considered. Second, evaluation criteria should be developed for each artistic and technical section, as in artistic gymnastics, which can result in a highly reliable judgement system. Additionally, based on the results of this study, it is suggested that performances should be evaluated using absolute and relative evaluation, respectively, for the technical and artistic sections. Although it is difficult to define the evaluation criteria of all the techniques in hip-hop dance because it is a freestyle dance displaying novelty and creativity, the basic techniques should be evaluated in the technical section based on their difficulty level and kinematic criteria to define the standard performance. In the artistic section, it might be useful to develop a judging support system that can minimise biases, such as facial expressions, physical features, shapes, and gender differences, which have been reported to influence evaluation (Cunningham et al., 1990; Pawlowski et al., 2000; Tovée et al., 1999). Future studies should identify the aesthetic factors that affect hip-hop dance evaluation. Third, while the World DanceSport Federation has already established a structured training and certification framework for breaking judges, such programmes need continuous evaluation and refinement, informed by empirical research on inter-rater reliability, to maintain judging transparency and fairness. Developing study materials similar to those used in other aesthetic sports (e.g., Fédération Internationale de Gymnastique, 2023) could further help judges, coaches, and competitors to understand and apply judging criteria consistently.

This study has several limitations. First, the analysis used relative evaluation values calculated by assigning a fixed weight of 20% to each category for the two competing performers; therefore, the results should be interpreted with caution when considering other weighting or ranking methods. Moreover, information on performance order (first or second dancer) was not available in the official data, and the same panel of judges evaluated all round-robin battles, making it difficult to assess potential order effects. Finally, this study relied on ICC and Kendall’s W to assess reliability. Future research could employ more advanced statistical approaches to partition variance components attributable to judges, dancers, or rounds and to further investigate the reliability of ranking outcomes in dance competitions.

## 5 Conclusion

This is the first study to investigate the reliability of the evaluation results of breakdancing as an Olympic sport. I found that the reliability of the judges’ scores was equivalent to that of DanceSport and hip-hop dance competitions and considerably lower than that of artistic gymnastics competitions. Additionally, unlike dance sports, hip-hop dance, and gymnastics competitions, which are judged through absolute evaluation, I found that the artistic category was more reliable than the technical category in breakdancing, which is assessed using relative evaluation. A reliable evaluation system is necessary for competitions wherein dance is performed as a sport, and the results will contribute to developing a more reliable evaluation system for hip-hop dance competitions. An objective evaluation system would also be useful for training dancers to achieve high scores from the judges in competitions.

## Data Availability

Publicly available datasets were analysed in this study. This data can be found here: https://olympics.com/en/paris-2024/results/breaking/b-boys/gpa-000100--.
