# Supplementary material for: Reliability of judging in Olympic breaking at the 2024 Paris games
Source: Front Psychol. 2025 Dec 3;16:1593158. doi: 10.3389/fpsyg.2025.1593158 (PMC12708583; doi:10.3389/fpsyg.2025.1593158)
Supplement: Supplementary file 1 [file Supplementary_file_1.docx]

Supplementary Table 1. Coefficients of reliability (ICC and Kendall’s W) for the five judging categories in the preliminary rounds.

| Category | ICC | | | | | | | | Kendall's W coefficient | |
| --- | --- | --- | --- | --- | --- | --- | --- | --- | --- | --- |
|  | Absolute agreement | | | | Consistency | | | |  | |
|  | Single | 95%CI | Average | 95%CI | Single | 95%CI | Average | 95%CI | W | p |
| Technique | 0.185 | 0.125–0.263 | 0.672 | 0.563–0.762 | 0.184 | 0.124–0.261 | 0.670 | 0.561–0.760 | 0.453 | 0.000 |
| Vocabulary | 0.326 | 0.252–0.415 | 0.814 | 0.752–0.865 | 0.324 | 0.250–0.413 | 0.812 | 0.750–0.864 | 0.500 | 0.000 |
| Originality | **0.471** | 0.391–0.560 | **0.889** | 0.853–0.920 | **0.469** | 0.389–0.557 | **0.888** | 0.851–0.919 | **0.610** | 0.000 |
| Execution | 0.322 | 0.248–0.411 | 0.809 | 0.748–0.863 | 0.320 | 0.246–0.409 | 0.809 | 0.746–0.862 | 0.526 | 0.000 |
| Musicality | 0.291 | 0.219–0.378 | 0.787 | 0.716–0.845 | 0.290 | 0.219–0.377 | 0.786 | 0.716–0.845 | 0.556 | 0.000 |

Bold values indicate the highest reliability coefficients (ICC and Kendall’s W) for the originality category.

Supplementary Table 2. Coefficients of reliability (ICC and Kendall’s W) for the five judging categories in the final rounds.

| Category | ICC | | | | | | | | Kendall's W coefficient | |
| --- | --- | --- | --- | --- | --- | --- | --- | --- | --- | --- |
|  | Absolute agreement | | | | Consistency | | | |  | |
|  | Single | 95%CI | Average | 95%CI | Single | 95%CI | Average | 95%CI | W | p |
| Technique | 0.255 | 0.162–0.381 | 0.755 | 0.634–0.847 | 0.251 | 0.159–0.376 | 0.751 | 0.629–0.844 | 0.355 | 0.000 |
| Vocabulary | 0.195 | 0.112–0.313 | 0.686 | 0.532–0.804 | 0.192 | 0.110–0.309 | 0.681 | 0.526–0.801 | 0.309 | 0.000 |
| Originality | **0.494** | 0.295–0.535 | **0.859** | 0.790–0.912 | **0.399** | 0.291–0.530 | **0.857** | 0.787–0.910 | **0.509** | 0.000 |
| Execution | 0.212 | 0.125–0.332 | 0.707 | 0.564–0.817 | 0.208 | 0.123–0.328 | 0.703 | 0.558–0.814 | 0.318 | 0.000 |
| Musicality | 0.235 | 0.145–0.359 | 0.735 | 0.604–0.834 | 0.231 | 0.143–0.354 | 0.731 | 0.599–0.832 | 0.354 | 0.000 |

Bold values indicate the highest reliability coefficients (ICC and Kendall’s W) for the originality category.
